# A 2-Tiered Rescue Protocol to Mitigate Difficulty-Based Failures of ChatGPT (GPT-5) and Gemini on the German M2 Medical Examination: Evaluation Study

**DOI:** 10.2196/86999

**Published:** 2026-07-21

**Authors:** Michael Constantin Kirchberger

**Affiliations:** 1 Department of Dermatology, Uniklinikum Erlangen, Deutsches Zentrum Immuntherapie, Friedrich-Alexander-Universität Erlangen-Nürnberg, Ulmenweg 18, Erlangen, 91054, Germany, 49 913135000; 2 Dermatology Center Ingolstadt, Ingolstadt, Germany

**Keywords:** large language models, LLM, medical education, medical examination, ChatGPT, Gemini, benchmark, difficulty, latency, artificial intelligence, AI

## Abstract

**Background:**

Large language models (LLMs) have demonstrated expert-level performance on medical licensing examinations, but most benchmarks focus on final accuracy. Critical gaps remain in understanding model efficiency (latency), the efficacy of tiered “rescue” protocols for error correction, and the systematic correlation between performance and human-rated question difficulty. The German M2 examination, paired with the AMBOSS platform’s user data–driven difficulty ratings, provides an opportunity to map AI performance against human cognitive load.

**Objective:**

This study aimed to move beyond singular accuracy scores by (1) evaluating and comparing the baseline (tier 1; T1) accuracy and response latency of next-generation rapid-response LLMs, (2) analyzing the efficacy of a 2-tiered rescue (tier 2; T2) protocol in correcting initial errors, and (3) correlating model performance with the user data–driven AMBOSS difficulty rating.

**Methods:**

We evaluated 4 LLMs (Gemini 2.5 Flash, Gemini 2.5 Pro, GPT-5 Instant, and GPT-5 Thinking) on the complete 316-item German M2 (Fall 2024) medical examination, including all multimodal (image-based) questions. A zero-shot copy-paste prompting strategy was used, and outputs were evaluated against ground-truth answers using a strict exact-match criterion. A 2-tiered protocol was used: T1 (Gemini Flash and GPT-5 Instant) provided baseline responses. If incorrect, a T2 (Gemini Pro and GPT-5 Thinking) model was deployed as a “rescue.” Performance was analyzed using the McNemar test, the Wilcoxon signed-rank test, the Fisher exact test, and logistic regression.

**Results:**

Baseline (T1) accuracy was identical at 91.5% (289/316; 95% CI 87.85%‐94.06%) for both Gemini 2.5 Flash and GPT-5 Instant, with 27 errors each. However, Gemini Flash (mean 1.57, SD 1.06 s) was significantly faster than GPT-5 Instant (mean 2.07, SD 1.89 s; *P*<.001). Additionally, GPT-5 Instant expended significantly more time on incorrect answers compared with correct ones (*P*=.002), whereas Gemini Flash showed no such hesitation (*P*=.81). The T2 rescue rate for GPT-5 Thinking (13/27, 48.2%; 95% CI 30.74%‐66.01%) was higher, though not statistically significant (*P*=.41), than that for Gemini 2.5 Pro (9/27, 33.3%; 95% CI 18.64%‐52.18%). This rescue protocol elevated final accuracy to 94.3% (298/316; 95% CI 91.18%‐96.37%) for the Gemini system and 95.6% (302/316; 95% CI 92.70%‐97.34%) for the GPT-5 system (*P*=.48). A strong, inverse relationship with difficulty was found: for every 1-point increase in difficulty, the odds of a correct T1 response decreased by 42.1% (odds ratio 0.579, 95% CI 0.425‐0.788; *P*<.001) for Gemini Flash and 47.7% (odds ratio 0.523, 95% CI 0.379‐0.720; *P*<.001) for GPT-5 Instant. This negative correlation persisted even after the rescue (*P*=.01 and *P*=.006, respectively).

**Conclusions:**

Expert-level LLM performance on the German M2 examination masks a critical vulnerability: a decrease in accuracy correlated with increased question difficulty. A 2-tiered “rescue” system is an effective strategy to mitigate these difficulty-based failures and achieve >95% accuracy.

## Introduction

The integration of large language models (LLMs) into medical education represents a significant technological shift, with profound implications for student learning and assessment [1]. Initial landmark studies demonstrated that general-purpose models, such as ChatGPT (OpenAI), could approach or exceed the 60% passing threshold for high-stakes medical licensing examinations, including all 3 steps of the United States Medical Licensing Examination (USMLE) [2]. This early *proof-of-concept* research sparked a wave of validation studies, highlighting the potential for LLMs to serve as educational aids but also revealing significant variability in their performance.

This initial research has been followed by a proliferation of benchmarks, necessitating systematic reviews to synthesize the evidence. These reviews confirm that LLM performance is highly heterogeneous, varying significantly based on model version, query language, and the specific examination source [3]. Performance on examinations in different countries has shown similar variability, often establishing a clear hierarchy between model generations. For instance, in the specific context of the German medical examination, previous research found that while GPT-4 achieved an expert-level 94.0% on the M2 medical examination, older models such as GPT-3.5 (76.4%) and Gemini Pro 2023 (Google LLC; 65.5%) performed substantially worse [4]. This fragmented landscape highlights that LLM performance is not a static metric but a moving target, highly dependent on the specific model and evaluation methodology [3]. Broader scoping reviews emphasize that while LLMs demonstrate immense potential for medical knowledge retrieval, their real-world application remains constrained by concerns over algorithmic hallucinations and inconsistent reasoning pathways across diverse clinical contexts. Consequently, researchers advocate for more nuanced evaluation frameworks that go beyond standardized multiple-choice metrics to capture the models’ reliability under varying cognitive loads [5]. Concurrently, research has expanded beyond general licensing examinations into specialty-specific fields, such as dermatology, to evaluate model performance on complex clinical decision-making tasks and visual case vignettes [6-8].

While recent benchmarks demonstrate that LLMs can achieve expert-level accuracy on medical examinations [3,4], this focus on singular accuracy scores obscures critical behavioral and operational characteristics. Key questions regarding model accuracy, the potential of multitiered rescue protocols, response latency, and robustness (performance correlation with human-rated difficulty) remain largely unaddressed.

To systematically investigate this vulnerability, the German M2 medical examination offers a unique advantage. Through the AMBOSS platform, each question is paired with a user data–driven difficulty rating, allowing us to map AI performance directly against human-perceived cognitive load. This methodology builds upon recent validation studies that directly compare generative AI accuracy against the empirical correct answer rates of human examinees, thereby exposing vulnerabilities tied to clinical cognitive load [9].

Addressing these difficulty-dependent failures requires moving beyond single-model paradigms. Real-world applications demand both speed and reliability, which necessitates the exploration of multitiered rescue protocols. Recent literature strongly advocates for the transition from isolated foundation models to compound AI systems, noting that single models are prone to silent reasoning failures when the clinical complexity exceeds their pretrained capacity [10]. By dynamically routing difficult queries to more intensive reasoning pathways, tiered systems attempt to bridge the critical gap between static benchmark success and reliable, safe clinical utility [11]. In such architectures, a rapid, efficient model handles standard queries, while a more computationally intensive reasoning model acts as a targeted safety net to correct errors on complex cases. Studies evaluating such clinical reasoning pathways indicate that while advanced computational methods significantly reduce error rates on complex tasks, they introduce efficiency and latency challenges that complicate their deployment as standalone, real-time solutions [12]. However, the exact mechanics, latency trade-offs, and true efficacy of such rescue systems remain largely unaddressed.

This study directly investigates these 3 gaps using a 2-tiered rescue methodology. The objectives are to (1) evaluate and compare baseline tier 1 (T1) accuracy and response latency, (2) analyze the efficacy of tier 2 (T2) rescue attempts in correcting initial T1 errors, and (3) correlate LLM model performance with the human-rated question difficulty.

## Methods

### Data Source and Question Set

The original questions, correct answers, and associated difficulty ratings for the fall 2024 written medical examination (M2) were extracted from the AMBOSS platform. All questions, including those incorporating images (eg, radiological scans and pathology slides), were included, resulting in a final analysis cohort of 316 items.

### Evaluation Protocol and Prompting

**Figure 1.**
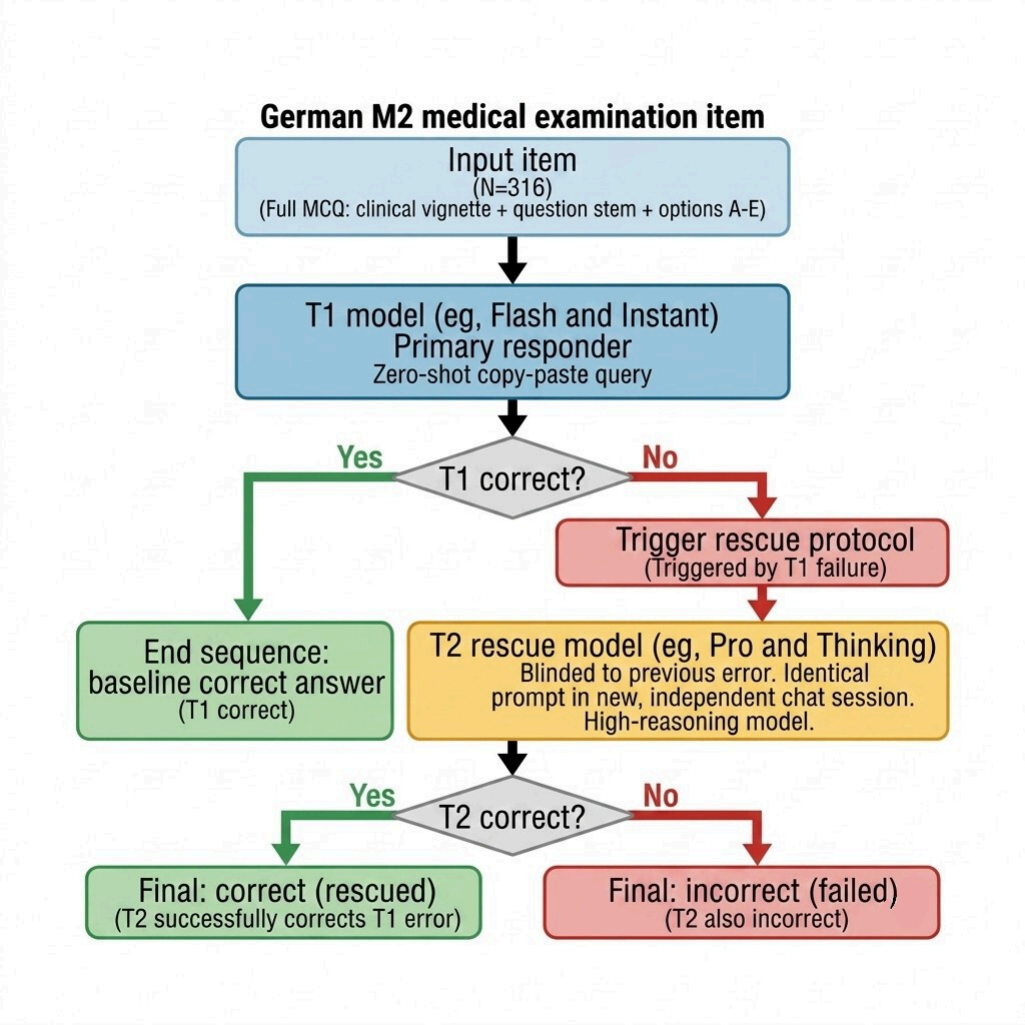
Operational flowchart of the 2-tiered rescue protocol. This cross-sectional evaluation study assessed large language models on the complete set of 316 single-best–answer questions from the German M2 medical licensing examination (fall 2024 cohort, sourced from the AMBOSS platform). In November 2025, all items were initially processed by a high-speed tier 1 (T1) model (Gemini 2.5 Flash or GPT-5 Instant) using a zero-shot prompt. Correct responses terminated the sequence. Incorrect responses triggered the rescue protocol, where the identical prompt was submitted to a high-reasoning tier 2 (T2) model (Gemini 2.5 Pro or GPT-5 Thinking) in a blinded, independent session to determine rescue efficacy. MCQ: multiple-choice question.

Four LLMs were evaluated, grouped into 2 systems with a 2-tiered rescue protocol (Figure 1): system 1 (Gemini 2.5 Flash as T1 and Gemini 2.5 Pro as T2) and system 2 (GPT-5 Instant as T1 and GPT-5 Thinking as T2). In this setup, a “2-tiered rescue protocol” is defined as a sequential inference architecture: a high-speed model (T1) acts as the primary responder for all queries, and a more advanced reasoning model (T2) is deployed exclusively to re-evaluate and correct the specific cases where T1 failed. All model interactions were conducted in November 2025.

To ensure methodological rigor and prevent context contamination, each question (whether single-item or case-based) was processed in a separate, new chat session. The model interaction simulated a zero-shot query. The German M2 medical examination is structured as a multiple-choice test, typically offering 5 distinct answer options (A-E) per question. The entire item, including the clinical vignette (if applicable), the specific question stem, and all answer options, was copied and pasted directly into the model’s interface. No additional comments or requests to format the output were provided. The German M2 examination consists exclusively of single-best-answer questions. Consequently, model outputs were evaluated against the official ground-truth answers using a strict exact-match criterion. An unambiguous selection of the single correct option was recorded as correct (value=1). Any other output—including selecting an incorrect option, selecting multiple options, or failing to make a definitive choice—was recorded as incorrect (value=0). No partial credit was awarded. Response latency was simultaneously recorded in seconds.

### Statistical Analysis

Performance metrics were compared at both the T1 baseline and the final, postrescue stage. Because all models were evaluated on the identical set of 316 examination items, the dataset is inherently paired at the item level. To compare the binary correctness (correct vs incorrect) between the 2 T1 models, and subsequently between the 2 final postrescue systems, the McNemar test was applied to these paired item-level outcomes. Differences in mean T1 response latency were evaluated using a paired 2-tailed *t* test, or the nonparametric Wilcoxon signed-rank test in the event of nonnormally distributed data. Rescue efficacy was defined as the proportion of initial T1 errors successfully corrected by the corresponding T2 model. The efficacy of these rescue attempts between the 2 T2 models was then compared using a chi-square test of independence, or Fisher exact test if contingency table sample sizes were small. Within-model latency differences between correct vs incorrect answers were assessed using the Mann-Whitney *U* test (2-sided). Normality was evaluated with the Shapiro-Wilk test.

To investigate the relationship between the ordinal difficulty rating and model accuracy, we used logistic regression. The correlation between difficulty and response latency was assessed using the Spearman rank correlation coefficient. *P*<.05 was considered statistically significant for all analyses.

### Question Difficulty

The primary difficulty metric was the AMBOSS “Hammer” rating, an ordinal scale ranging from 1 (easiest) to 5 (hardest). Unlike static or subjective expert assessments, this rating is a dynamic, user data–driven metric generated by the platform based on the aggregate performance of tens of thousands of medical students preparing for the examination. For example, a 1-Hammer question is answered correctly by the vast majority of users, whereas a 5-Hammer question is answered correctly by only a small fraction. Because this metric is continuously calibrated against the actual success rates of the target demographic, it provides a highly robust, ecologically valid proxy for human-perceived cognitive load and clinical complexity.

### Ethical Considerations

This study used publicly available examination data and user-generated difficulty ratings from the AMBOSS platform. The research did not involve human participants, and all data were anonymized. As such, in accordance with Section 15 of the Professional Code of Conduct of the Bavarian State Medical Association (BLÄK)[13], formal institutional review board consultation was not required, as research projects using strictly anonymized data are exempt.

## Results

### Baseline (T1) Performance: Accuracy and Latency

Analysis of the 316 examination items revealed identical baseline accuracy for both T1 models. Gemini 2.5 Flash and GPT-5 Instant both correctly answered 289 (91.5%) questions, achieving an accuracy of 91.5% (95% CI 87.85%-94.06%) and committing 27 (8.5%) errors each.

However, a paired comparison highlighted that the error profiles were not identical. The models only shared 16 (5.1%) incorrect answers. An analysis of these 16 shared errors showed a median difficulty of 3.0 (IQR 3.0-4.0), with 43.8% (7/16) rated as difficulty 4 or 5.

There were 22 discordant items: Gemini 2.5 Flash answered 11 (50%) questions incorrectly that GPT-5 answered correctly, while GPT-5 answered 11 (50%) different questions incorrectly that Gemini answered correctly. As these discordant pairs were equal (11 vs 11), McNemar test showed no statistically significant difference in the overall error rates between the 2 models (*P*=.999).

**Figure 2.**
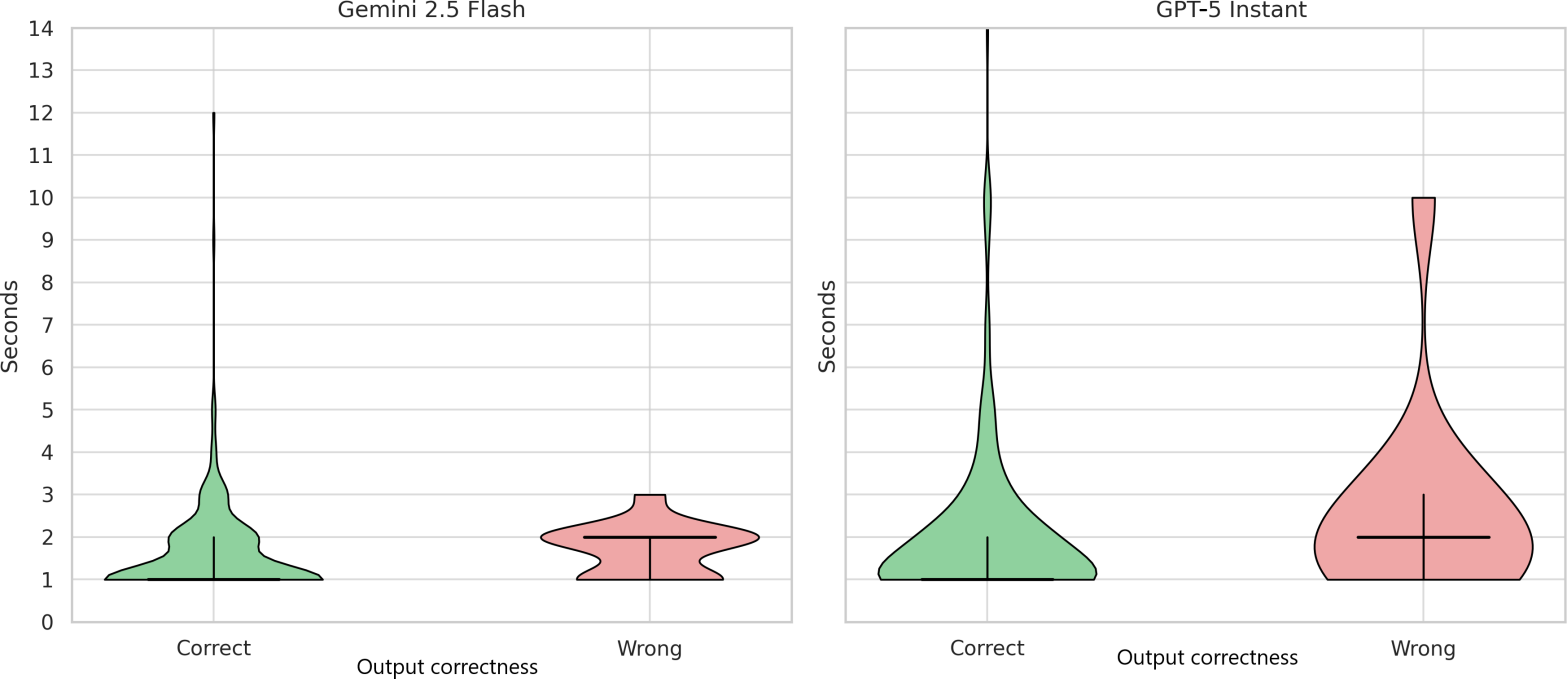
Tier 1 response latency distribution. Comparison of response latencies (in seconds) for Gemini 2.5 Flash and GPT-5 Instant. Data were generated during a cross-sectional evaluation of the complete German M2 medical licensing examination (fall 2024 cohort; N=316 items) conducted in November 2025. The central mark indicates the median, and the box edges represent the IQR. The latency for Gemini 2.5 Flash was statistically significantly lower than that for GPT-5 Instant (Wilcoxon signed-rank test; *P*<.001).

Significant differences were observed in response latency (Figure 2). The Gemini 2.5 Flash model (mean 1.57, SD 1.06 s) was significantly faster than the GPT-5 Instant model (mean 2.07, SD 1.89 s). Shapiro-Wilk tests indicated that the latency data were nonnormally distributed (*P*<.001 for both). Therefore, the nonparametric Wilcoxon signed-rank test was used (as specified in the Methods section), confirming that this difference in latency was statistically significant (*P*<.001).

### Efficacy of T2 Rescue Attempts

Of 316 items, the analysis of rescue efficacy focused on the 27 (8.5%) incorrect responses generated by the Gemini 2.5 Flash (T1) model and the 27 (8.5%) incorrect responses generated by the GPT-5 Instant (T1) model.

The T2 rescue model, Gemini 2.5 Pro, successfully corrected 9 (33.3%) of its T1 errors, achieving a rescue rate of 33.3% (95% CI 18.64%-52.18%). The corresponding T2 model, GPT-5 Thinking, successfully corrected 13 (48.2%) of its T1 errors, resulting in a rescue rate of 48.2% (95% CI 30.74%-66.01%; ). A Fisher exact test was used to compare these 2 proportions; the difference in rescue efficacy between the 2 T2 models was not statistically significant (*P*=.41).

Following the application of the 2-tiered rescue protocol, the final accuracy for the Gemini system (Flash and Pro) was 94.3% (298/316; 95% CI 91.18%-96.37%). The final accuracy for the GPT-5 system (Instant and Thinking) was 95.6% (302/316; 95% CI 92.70%-97.34%).

To compare the final paired outcomes of the 2 complete systems, McNemar test was performed. The analysis identified 18 discordant pairs: in 11 (61.1%) cases, the GPT-5 system was correct while the Gemini system was incorrect, and in 7 (38.9%) cases, the Gemini system was correct while the ChatGPT system was incorrect. This difference in final system-level accuracy was not statistically significant (*P*=.48).

### Performance in Relation to Question Difficulty

**Figure 3.**
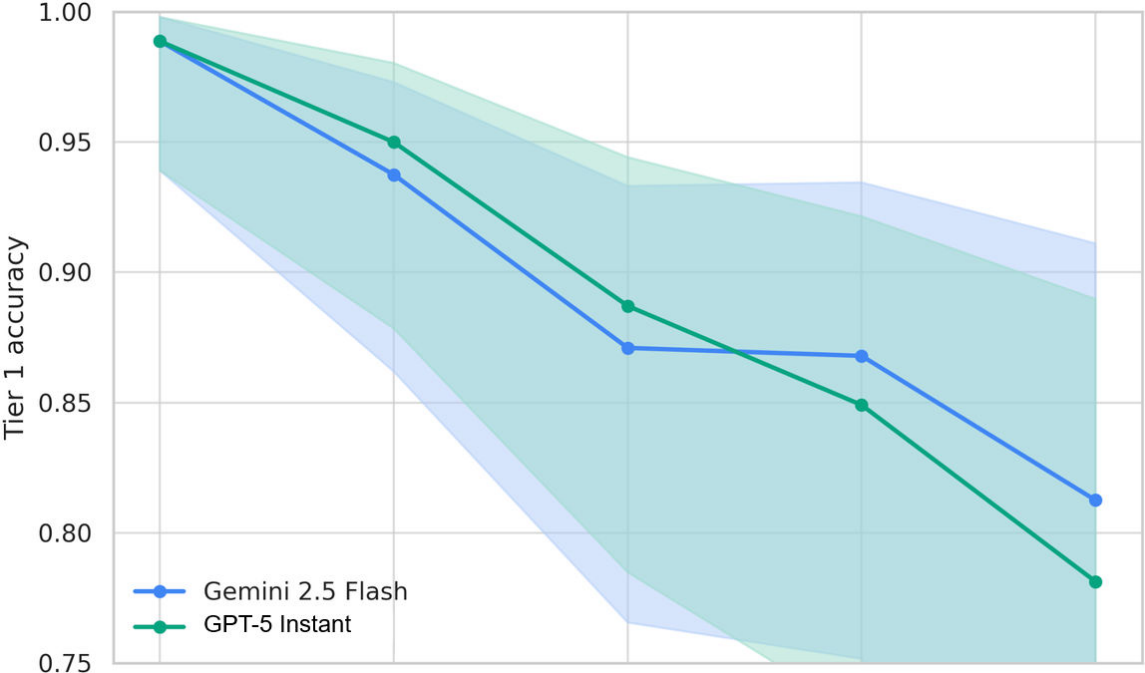
Tier 1 (T1) model accuracy in relation to question difficulty. Baseline (T1) accuracy for Gemini 2.5 Flash and GPT-5 Instant across 5 levels of human-rated question difficulty. The dataset comprised 316 items from the fall 2024 German M2 medical examination, with difficulty ratings (1=easiest to 5=hardest) derived from the AMBOSS platform user data. In this cross-sectional analysis conducted in November 2025, both models demonstrated a statistically significant decrease in accuracy as human-perceived question difficulty increased (logistic regression, *P*<.001 for both models). Logistic regression confirmed that this negative trend was statistically significant for both T1 models. For every 1-point increase on the 5-point difficulty scale, the odds of a correct T1 response decreased by 42.1% for Gemini Flash (odds ratio [OR] 0.579, 95% CI 0.425-0.788; *P*<.001) and by 47.7% for GPT-5 Instant (OR 0.523, 95% CI 0.379-0.720; *P*<.001). This significant negative association persisted even after the T2 rescue attempts. The odds of a correct final (after rescue) response still decreased significantly for both the Gemini system (OR 0.633, 95% CI 0.442-0.909; *P*=.01) and the GPT-5 system (OR 0.550, 95% CI 0.360-0.840; *P*=.006).

A clear inverse relationship was observed between question difficulty and model accuracy. Descriptively, T1 accuracy for Gemini Flash decreased from 98.9% (difficulty 1) to 81.3% (difficulty 5). Similarly, GPT-5 Instant accuracy fell from 98.9% (difficulty 1) to 78.1% (difficulty 5; Figure 3).

The relationship between difficulty and T1 response latency was less pronounced. A Spearman rank correlation test found no statistically significant association for Gemini Flash (*P*=.19). For GPT-5 Instant, a weak but statistically significant positive correlation was detected (*P*=.004), indicating that it tended to take slightly longer to answer more difficult questions.

## Discussion

### Principal Findings

This study evaluated the baseline performance of rapid-response LLMs, the efficacy of a 2-tiered rescue protocol, and the impact of question difficulty. We found that while these models achieve expert-level accuracy with distinct latency and error profiles, a hierarchical rescue system effectively mitigates systematic reasoning failures, although overall performance remains significantly constrained by task complexity.

Our study’s baseline finding of 91.5% (289/316) accuracy (95% CI 87.85%-94.06%) for both Gemini 2.5 Flash and GPT-5 Instant confirms that expert-level performance is the new standard for flagship models on the German M2 medical examination. These results are situated in the context of research that evaluated earlier model iterations on a similar examination. The investigators reported that while the full GPT-4 model achieved 94.0% accuracy on the M2, older models such as GPT-3.5 and Gemini Pro lagged significantly at 76.4% and 65.5%, respectively [4].

Crucially, our study’s cohort of 316 items included questions with integrated images (eg, radiological scans and pathology slides), unlike various previous studies. Our 91.5% baseline therefore reflects a more holistic assessment of the models’ multimodal capabilities in a true-to-life examination context, aligning with recent studies on USMLE image questions that also report high accuracy [14] and should be incorporated in future research as LLMs advance in the assessment of images [15,16].

This comparison provides a critical nuance. Our 91.5% accuracy was achieved by next-generation models explicitly optimized for speed (Gemini Flash and GPT-5 Instant). This baseline is substantially higher than that of all previous-generation models except the full-capacity GPT-4, against which our models show only a minor 2.5 percentage point deficit. This suggests a potential trade-off between the new generation’s rapid-response models and the previous generation’s peak-performance model.

However, as the field moves past simple accuracy scores converging above 90% [17], our study provides critical behavioral insights that prior studies on the German examination did not explore. Although their accuracy was identical, the T1 models displayed starkly different underlying behaviors. Notably, their error profiles diverged substantially, with only 16 incorrect items overlapping. This demonstrates that quantitative equivalence does not guarantee qualitative equivalence in clinical reasoning; each model exhibits idiosyncratic blind spots. Such divergence reinforces the limitations of singular accuracy metrics and further justifies the use of tiered architectures, where an independent secondary model can mitigate these model-specific failures. A significant efficiency difference was observed: Gemini 2.5 Flash (mean 1.57, SD 1.06 s) was 24.1% faster than GPT-5 Instant (mean 2.07, SD 1.89 s), a statistically significant gap (*P*<.001). Furthermore, the models exhibited fundamentally different failure patterns. GPT-5 Instant expended significantly more time (median 2.00 s, IQR 1.00-3.00 s) on the 27 items it answered incorrectly than the 289 items it answered correctly (median 1.00 s; IQR 1.00-2.00 s, *P*=.002). In sharp contrast, Gemini 2.5 Flash’s latency for incorrect answers (median 1.00 s, IQR 1.00-2.00 s) was indistinguishable from its correct answers (*P*=.81), resembling a rapid, confidently wrong retrieval error. Therefore, while our accuracy findings confirm the expert-tier performance reported on previous examinations [4], our latency and failure-mode analyses demonstrate that a simple accuracy score is an insufficient proxy for a model’s cognitive process and real-world utility.

### Rescue Efficacy and the Persistent Challenge of Question Difficulty

The implementation of a 2-tiered rescue system proved to be an effective strategy, elevating the final system-wide accuracy to 94.3% (298/316) for the Gemini system and 95.6% (302/316) for the ChatGPT system. This postrescue performance is a key finding. Our ChatGPT system’s 95.6% and our Gemini system’s 94.3% are both comparable to, or slightly exceed, the 94.0% peak performance of the full-capacity GPT-4 [4]. This demonstrates that a hybrid approach, using a fast T1 model for most queries and a more powerful T2 model for error correction, can achieve the same peak accuracy as the previous generation’s best-performing, full-capacity model.

However, the necessity of this rescue system highlights the most significant finding of our study: the pervasive impact of question difficulty. The 8.5% (27/316) of errors made by the T1 models were not random. As confirmed by our logistic regression, the odds of a correct T1 response decreased by 42% to 48% for every 1-point increase in difficulty (*P*<.001 for both models). This finding quantifies a phenomenon seen in other studies, which found that models excel at lower-order recall but fail at higher-order thinking [8]. This vulnerability persisted even after the rescue, as the 16 items failed by both T1 models (median difficulty 3, IQR 3.0-4.0) represented the hard ceiling of T1 models.

Crucially, the T2 models differed in their ability to break this hard ceiling. While the overall rescue rates (9/27, 33.3% for Gemini Pro 2.5 vs 13/27, 48.2% for GPT-5 Thinking) were not statistically different (*P*=.41), the GPT-5 Thinking system’s superior rate was descriptively driven by its success on these high-difficulty items (rescuing 63.6% of level 4 or 5 errors, vs 30% for Gemini Pro 2.5). This suggests that GPT-5 Thinking (T2) may be more adept at resolving the complex reasoning failures of its Instant (T1) counterpart. This aligns with multimodal studies finding that while models achieve high accuracy on image-based questions, their incorrect answers often contain inference errors and image misunderstandings [10,16].

### Implications for Medical Education and Practice

The findings have direct implications for the integration of LLMs into medical education, a topic of intense discussion. Our study serves as a critical caveat to the enthusiasm surrounding expert-level scores. The primary risk identified is the significant, inverse relationship between accuracy and question difficulty. Students are most likely to consult an LLM for topics they find challenging, precisely the level 4 and 5 questions where our T1 models’ failure rates (6/32, 18.7% and 7/32, 21.9%, respectively) were highest. This creates a high-risk scenario for reinforcing plausible but wrong information.

However, our 2-tiered methodology suggests a practical framework for mitigating this risk. The data support a hierarchical implementation strategy: fast, efficient T1 models (eg, Gemini 2.5 Flash, given its 24.1% speed advantage) could be used for high-volume, lower-stakes queries. For critical queries or those identified as high difficulty, our results demonstrate the value of an integrated rescue step. The GPT-5 system’s ability to achieve 95.6% (302/316) final accuracy, driven by its 63.6% success rate in rescuing level 4 or 5 errors, suggests that this approach can provide a safety net, bringing overall performance to the level set by the full GPT-4 model [4].

### Limitations and Future Directions

Our protocol used a zero-shot copy-paste method to simulate naive user behavior. While this reflects a realistic use case, the literature indicates that advanced prompt-engineering strategies, such as chain-of-thought prompting, can further improve accuracy [17]. Additionally, this study used a single-run evaluation per item. While reflecting a standard user query, it does not account for the stochastic variability of LLM outputs across repeated samplings. Qualitative analysis of the 16 dual-model failures indicates that errors were primarily driven by reasoning bottlenecks in multistep clinical scenarios and negative-selection questions, rather than a lack of underlying medical knowledge.

Future research should prioritize 2 areas. The most critical next step is a qualitative analysis of the shared errors and the final unrescued errors. We know these items are difficult (median 4.0), but we do not know why. Investigating whether these errors stem from failures in causal reasoning, managing ambiguity, or accessing niche knowledge is essential. On the basis of our findings, we propose 2 practical principles for autonomous tiered routing: First, latency and confidence triggers: systems can leverage internal model metrics, such as confidence scores or real-time processing latency. Our novel finding that GPT-5 Instant hesitated significantly on incorrect answers (*P*=.002) suggests that algorithmic “hesitation” is a viable, real-time trigger for T2 escalation. Second, heuristic complexity filtering: routing mechanisms can analyze structural prompt characteristics, such as word count or the presence of multistep clinical vignettes to automatically bypass T1 for highly complex queries, effectively operationalizing our rescue framework for clinical practice.

### Conclusions

In conclusion, this study confirms that next-generation LLMs achieve expert-level baseline performance on the German M2 medical examination. However, our research demonstrates the need to move beyond standard accuracy metrics. By mapping AI performance directly against human cognitive load rather than evaluating single models in isolation, we uncovered hidden latency patterns and difficulty-dependent failure modes, revealing divergent model behaviors. To address these systematic errors, we validated a novel 2-tiered rescue protocol. We demonstrated that this hierarchical architecture effectively mitigates such failures, elevating final accuracy to more than 95%. Ultimately, these findings show that relying on a single model is insufficient for real-world applications. Ensuring the safe and effective integration of AI into medical education and clinical environments requires hierarchical systems that can explicitly manage varying levels of query difficulty.

## Abbreviations

LLM: large language model
T1: tier 1
T2: tier 2
USMLE: United States Medical Licensing Examination
